# Zebrafish Rab5 proteins and a role for Rab5ab in nodal signalling

**DOI:** 10.1016/j.ydbio.2014.11.007

**Published:** 2015-01-15

**Authors:** Emma J. Kenyon, Isabel Campos, James C. Bull, P. Huw Williams, Derek L. Stemple, Matthew D. Clark

**Affiliations:** a Wellcome Trust Sanger Institute, Wellcome Trust Genome Campus, Hinxton, Cambridge CB10 1SA, United Kingdom; b Sequencing Technology Development, The Genome Analysis Centre, Norwich Research Park, Colney, Norwich NR4 7UH, United Kingdom; c Champalimaud Centre for the Unknown, Fundação Champalimaud, Lisboa, Portugal; d Department of Biosciences, Swansea University, Swansea SA2 8PP, United Kingdom

**Keywords:** Rab5, Zebrafish, Nodal

## Abstract

The *RAB5* gene family is the best characterised of all human *RAB* families and is essential for *in vitro* homotypic fusion of early endosomes. In recent years, the disruption or activation of Rab5 family proteins has been used as a tool to understand growth factor signal transduction in whole animal systems such as *Drosophila melanogaster* and zebrafish. In this study we have examined the functions for four *rab5* genes in zebrafish. Disruption of *rab5ab* expression by antisense morpholino oligonucleotide (MO) knockdown abolishes nodal signalling in early zebrafish embryos, whereas overexpression of *rab5ab* mRNA leads to ectopic expression of markers that are normally downstream of nodal signalling. By contrast MO disruption of other zebrafish *rab5* genes shows little or no effect on expression of markers of dorsal organiser development. We conclude that *rab5ab* is essential for nodal signalling and organizer specification in the developing zebrafish embryo.

## Highlights

• We have examined the activities of each of the zebrafish Rab5 genes using morpholino knockdowns.
• Loss of one Rab5 isoform, Rab5ab, affects formation of the dorsal organizer.
• Rab5ab overexpression leads to ectopic expression of dorsal markers.

## Introduction

Cellular motility and cohesion are essential processes in vertebrate early embryonic development. Integral to the processes are intracellular trafficking events, which direct the signalling between cells and the movement and adhesion of cells. Intracellular signalling is, in turn, heavily dependent on vesicle transport events, under the control of RAB proteins, which localise to specific intracellular compartments and pilot vesicles to target membranes (Zerial and McBride, 2001).

The RAB family of small GTPase enzymes is the largest sub-family in the Ras super-family. RABs are found in all eukaryotes, with 64 (including 4 pseudogenes) RAB genes present in the human reference genome (Zerial and McBride, 2001; Colicelli, 2004; Seal et al., 2011). The current thinking is that five core RABs are required for the basic functions of a cell, RAB1, RAB5, RAB6, RAB7 and RAB11 (Chavrier et al., 1990; Bucci et al., 1992; Pereira-Leal and Seabra, 2001). The RAB5 family is perhaps the best characterised of all 43 human RAB families and its members have been shown to localize to clathrin-coated vesicles, early endosomes and the plasma membrane (Bucci et al., 1992). The proteins are essential for *in vitro* homotypic fusion of early endosomes and are able to increase the rate of endocytosis *in vivo* when overexpressed (Gruenberg and Howell, 1989; Gorvel et al., 1991; Li and Stahl, 1993).

For cell and developmental biology much interest in Rab5 activity has resulted from their use as a tool to alter endocytosis. For example, activation and disruption of Rab5 proteins have been used to understand cell movements during gastrulation and how signalling factors move though a developing embryo (Scholpp and Brand, 2004; Ulrich et al., 2005; Hagemann et al., 2009; Tay et al., 2010; Torres and Stupack, 2011). Similarly, Rab5 proteins have also been used to understand human diseases such as Alzheimer׳s disease (Ginsberg et al., 2010) and the motility and invasiveness of tumour cells (Torres and Stupack, 2011).

There are three mammalian RAB5s: RAB5A, RAB5B and RAB5C, which have been studied in mammalian cell culture assays (Wilson and Wilson, 1992; Singer-Kruger et al., 1994). Normally, such studies do not distinguish individual RAB5 gene activities (Bernard et al., 2010; Hagiwara et al., 2011), though other cell-based studies have shown that each member of the family can differentially regulate trafficking (Baskys et al., 2007; Chen et al., 2009). For example, RAB5 proteins are differentially recognised by different kinases (Chiariello et al., 1999). RAB5A is efficiently phosphorylated by extracellular-regulated kinase 1 but not by extracellular-regulated kinase 2, while cdc2 kinase preferentially phosphorylates Ser-123 of RAB5B. It was suggested that phosphorylation could be important to differentially regulate the function of the RAB5 isoforms (Chiariello et al., 1999).

In whole-animal studies of development, Rab5 family proteins have been used as a tool to understand trafficking of growth factors and their signals (Scholpp and Brand, 2004; Ulrich et al., 2005; Hagemann et al., 2009). In these studies, however, individual family members are rarely distinguished and sometimes used interchangeably. Given that cell culture studies have suggested divergent roles for the various *rab5* genes (Baskys et al., 2007; Chen et al., 2009), one aim of this study was to assay *in vivo* for differing roles of individual members of the *rab5* gene family during early embryonic development. Moreover, we sought to understand how these effects are manifest in the dynamically developing embryo, rather than as isolated signalling events, with the overall aim of understanding how individual *rab5* family genes contribute to zebrafish early developmental events such as dorsal organiser specification.

## Materials and methods

### Probe synthesis and *in situ hybridisation*

Whole-mount *in situ* hybridisation was carried out essentially as described by Thisse and Thisse (2008). The *rab5aa* gene probe was transcribed directly from cDNA clone IMAGp998K098962Q (Source BioScience) and linearized with Sal1 (NEB) and transcribed with SP6 RNA polymerase (NEB). Embryos were manually dechorionated and fixed in 4% paraformaldehyde at 24 h post fertilisation (hpf).

### 5′ capped RNA synthesis

Capped *rab5ab* RNA was synthesised *in vitro* using 5 µg (5 µl) of linearized *rab5ab* DNA (IRAKp961M19104 sub-cloned into a pCS2+ vector or a GFP-pCS2+vector) in a 50 µl reaction containing 10 µl of 5× transcription buffer, 5 µl of 0.1 M DTT, 5 µl of 5 mM CAP (NEB), 5 µl of 1 mM GTP (NEB), 5 µl of 5 mM UTP (NEB), 5 µl of 5 mM ATP (NEB), 5 µl of 5 mM CTP (NEB), 2 µl of RNAse inhibitor (NEB) and 3 µl of SP6 RNA polymerase (NEB) incubated at 37 °C for 20 min when 4 µl of 10 mM GTP was added and incubated at 37 °C for a further 2 h. An additional 3 µl of RNAse free DNAse (Promega) was added and the reaction incubated at 37 °C for a further 20 min. The RNA was separated from the other reaction components using Chroma-100 spin columns (Clontech).

### MO injections

The following MOs (Genetools) were used in this study:

*rab5aa* MO 5′-GACAGTTGTCAATCACCCCGTCTTC,

*rab5ab* MO 5′-TCGTTGCTCCACCTCTTCCTGCCAT,

*rab5ab* MO2 5′-GACCCAAAACCCCAATCTCCTGTAC,

*rab5ab* (MM) 5 bp mismatch 5′-GACgCAAAAgCCgAATCTgCTcTAC,

*rab5ab* splice MO: 5′-ATGAAGCGTTTGTCTTACCTCCTAT

*rab5b* MO 5′-CCTGCCTGTCCCACGGGTACTCATG,

*rab5c* MO 5′-CGCTGGTCCACCTCGCCCCGCCATG,

Standard Control MO 5′-CCTCTTACCTCAGTTACAATTTATA.

*p53* MO: GCGCCATTGCTTTGCAAGAATTG

Oligonucleotides were diluted in MO buffer (5 mg/ml phenol red (Sigma), 4 mM HEPES pH 7.2 (Sigma), 160 mM KCl (Sigma)) and 1.4 nl of MO solution was injected into the yolk of the 1–4 cell stage embryo. We used 10 ng of *rab5aa* MO, 3 ng or 5 ng of *rab5ab* MO, 8 ng for *rab5b* MO, 6 ng for *rab5c* MO and those quantities with an additional 2 ng for standard control MO injections.

### Electron microscopy

Whole zebrafish embryos were dechorionated manually and fixed overnight with 2% glutaraldehyde, 2% paraformaldehyde in 0.1 M sodium cacodylate buffer (pH 7.2) (SCB). The following day, embryos were washed for 10 min in SCB and post-fixed for 1 h in 1% osmium tetroxide in SCB. They were washed again with SCB and stained *en bloc* with 1% aqueous uranyl acetate for 1 h. The samples were then dehydrated through a graded ethanol series, followed by two changes of propylene oxide over 20 min and embedded in Epon resin (Agar Scientific). We then cut 50 nm ultra-thin sections, mounted them on pioloform coated slot grids and stained with 1% aqueous uranyl acetate for 15 min, followed by Reynold׳s lead citrate for 7 min. Sections were visualised in a Jeol 1200 EX electron microscope.

### Epiboly movement assay

Embryos were dechorionated at dome stage, 30% epiboly or shield stage then placed in glass dishes containing 5 mg/ml of biotinylated-dextran (Molecular probes 10,000 mw lysine fixable) in 1× Danieau solution for 20 min (Shih and Fraser, 1996). Embryos were then washed and fixed in 4% paraformaldehyde solution. Fixed embryos were dehydrated in methanol then rehydrated in PBT (10 mM phosphate buffered saline, 0.05% Tween-20, pH 7.4) and incubated for 30 min in 1:5000 horseradish peroxidase-labelled streptavidin in PBT. Embryos were washed three times with PBT and soaked for 30 min in DAB/PBT (0.4 mg/ml 3,3′-Diaminobenzidine in PBT). The solution was then changed for staining solution, DAB/PBT with 0.003% H_2_O_2,_ and examined as reaction product developed over 30 min. Once stained the reaction was stopped by rinsing with PBT.

### RT-PCR

RNA was isolated from control and *rab5ab* MO injected embryos using Trizol as per manufacturers protocol (Invitrogen). We used 1.5 μg of RNA to produce cDNA by reverse transcription (Superscript III, Life Technologies). Quantitative PCR was performed using Applied Biosystems TaqMan Universal PCR Master Mix and TaqMan primers for *ndr1*, *gsc*, *ntl, chd*, *tfr1b, wnt8a, bmp2b* and *bmp4* designed and made by Applied Biosystems on an ABI prism using 7000 system software. Each of the cDNAs, *ndr1*, *ntl*, *gsc*, *chd*, *tfr1b*, *wnt8a, bmp2b* and *bmp4* were analysed separately. Preliminary inspection supported normalisation of the cycle threshold (Ct) data transformation by 1/log_2_(Ct) to stabilise variances and allow intuitive visualisation using box-whisker plots. Such a transformation allows intuitive interpretation of changes in gene expression (a difference of one equates to a two fold change in expression), with further reciprocal transformation stabilising the mean-variance relationship (Eastwood et al., 2008; Bull et al., 2012). Expression of *tfr1b* was shown not to change between control and MO injected embryos (L.R.=0.097, *p*=0.76) but did change between stage (L.R.=13.4, *p*<0.001, 30% v shield stage) therefore all other genes were normalised to *tfr1b* within stage. The transformed data were analysed using linear mixed-effects models (Pinheiro and Bates, 2000) with MO-injected *versus* control embryos as a categorical fixed effect and the experimental design captured as hierarchical random effects, with technical replication (RNA preparations) nested within biological replication (individual embryos). Hypotheses on the effects of MO injections were tested using likelihood ratios, with the test statistic assumed to be Chi-squared distributed (Pinheiro and Bates, 2000). R software was used for calculations and graph generation (R Core Development Team, 2008).

### Phalloidin labelling

At 48 h post fertilisation (hpf) zebrafish embryos were fixed in 4% PFA overnight. Embryos were washed in PBS-triton and incubated in the dark with gentle agitation at room temperature overnight in 2.5 ug/ul Rhodamine phalloidin (Molecular Probes R415). Embryos were repeatedly washed in PBS-tween and imaged on an agarose plate using a Nikon SMZ800 fluorescent stereomicroscope and camera.

## Results

### Identification of zebrafish homologues of Human RAB5A, RAB5B and RAB5C

**Fig. 1:**
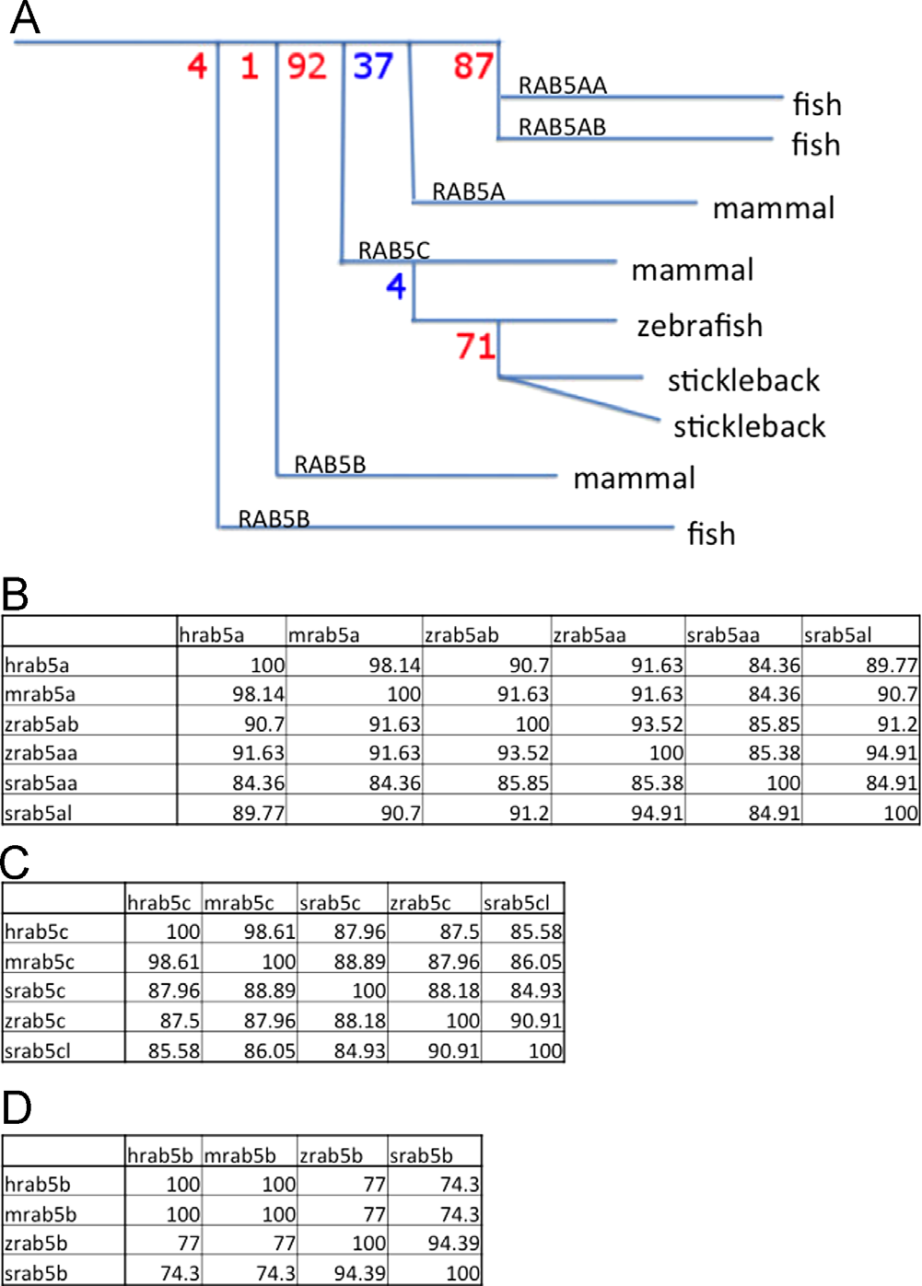
*rab5* family shows conservation between species (A) Stylised version of the RAB5 family tree constructed using Genomicus version 61.01 (Muffato et al., 2010). Bootstrap (%) values are shown for tree nodes; red=gene duplication (paralogues); blue=speciation (orthologues). (B) Conservation of RAB5A protein sequence between human, mouse, zebrafish and stickleback. (C) Conservation of RAB5B protein sequence between human, mouse, zebrafish and stickleback. (D) Conservation of RAB5C protein sequence between human, mouse, zebrafish and stickleback. Conservation percentage identity matrix produced by Clustal Omega (Sievers et al., 2011).

The three RAB5 genes in humans were used as query sequence to identify zebrafish Ensembl predictions (Ensembl Zebrafish 19.3.2) (Flicek et al., 2011), which was based on the zebrafish genome assembly (Zv3)(Howe et al., 2013), mRNAs and ESTs using WU-BLAST followed by MSPcrunch filtering (Sonnhammer and Durbin, 1994). We used a threshold of 60% identity as a human to zebrafish match. Zebrafish EST sequences were retrieved along with capillary sequence traces (http://www.ncbi.nlm.nih.gov/Traces/), if available, which were quality clipped and vector masked with PreGap (Bonfield and Staden, 1996). These were then used to assemble cDNAs and ESTs in Gap4 (Bonfield et al., 1998) giving a total of four zebrafish *rab5* genes with recent genome assembly׳s not revealing any further orthologs. These four *rabs* are currently known as *rab5aa* (ENSDARG00000018602, ZDB-GENE-030131-139), *rab5ab* (ENSDARG00000007257, ZDB-GENE-040122-3), *rab5b* (ENSDARG00000016059, ZDB-GENE-040426-2593) and *rab5c* (ENSDARG00000026712, ZDB-GENE-031118-30). The *rab5ab* gene generates at least two coding variants while *rab5aa, rab5b* and *rab5c* have only one annotated coding variant each. Using Genomicus version 61.01 (Muffato et al., 2010) and confirmed by Ensembl release 62 we find that the *rab5a* duplication is likely to have resulted from the teleost specific whole-genome duplication, as similarly duplicated *rab5a* genes are also present in the *Tetraodon*, Medaka and Stickleback genomes (Fig. 1).

### Morphological loss of function screen of *rab5* family

To determine whether the *rab5* genes in the zebrafish genome have similar functions, we knocked down each individually, using MOs targeting the ATG start codon followed by morphological phenotyping of the MO injected embryos (Nasevicius and Ekker, 2000).

#### rab5aa

**Fig. 2:**
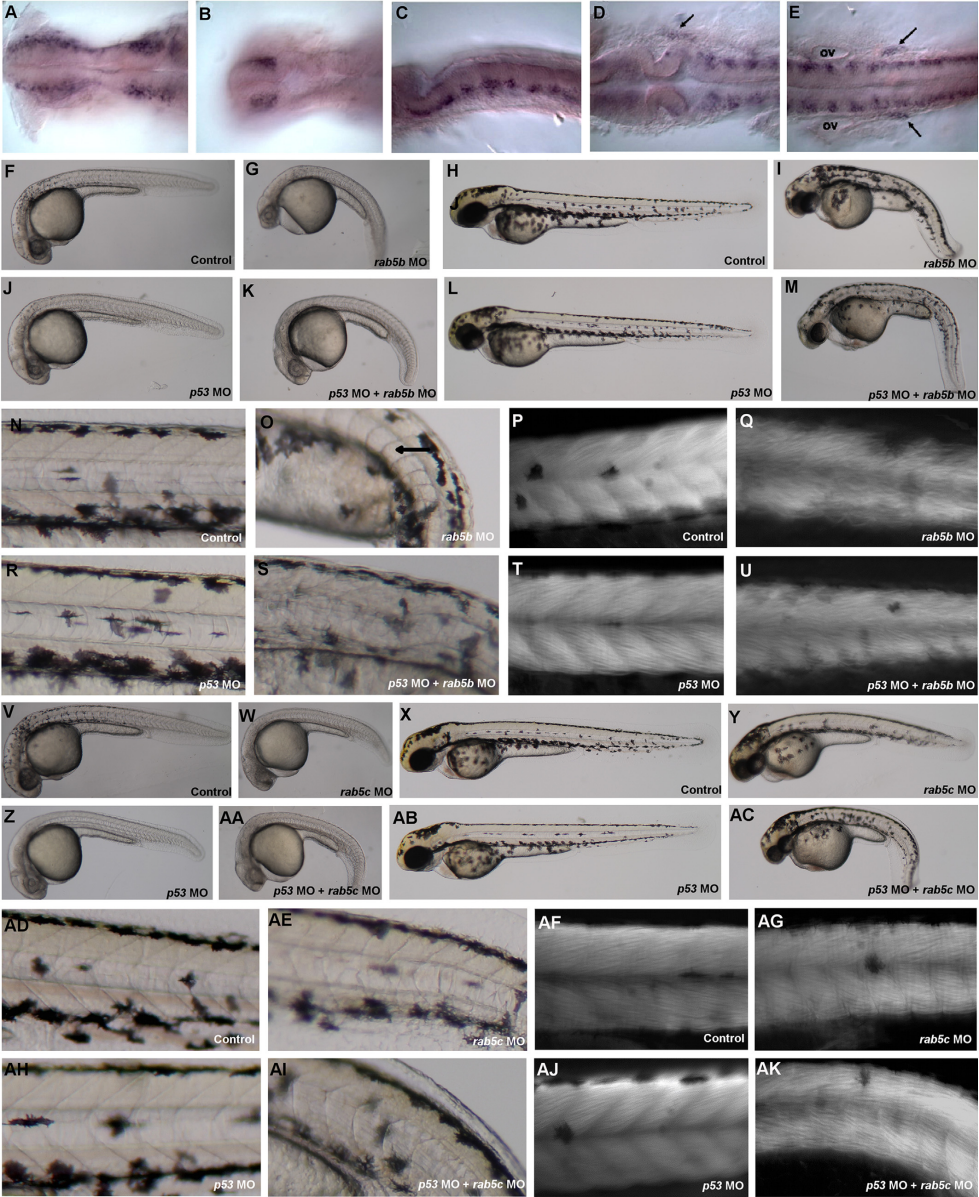
Expression and loss of function of the *rab5a* family. (A) Expression of *rab5aa* in the forebrain and midbrain region of a 24 hpf embryo. (B) Forebrain region with dorsal focus showing two patches of bilateral telencephalic cells. (C) Hindbrain region showing expression on the central region of each rhombomere. (D) Expression of *rab5aa* in cells outside the neural tube at the level of the midbrain/hindbrain boundary (arrow). (E) Expression of *rab5aa* at the end of the hindbrain (arrows) and in the trunk of the embryo. (F) Side view of a 24 hpf embryo injected with 10 ng of control MO (*n*=205/207). (G) Side view of a 24 hpf embryo injected with 8 ng of *rab5b* MO (*n*=141/143). (H) Side view of a 48 hpf embryo injected with 10 ng of control MO (*n*=204/204). (I) Side view of a 48 hpf embryo injected with 8 ng of *rab5b* MO (*n*=117/126). (J) Side view of a 24 hpf embryo injected with 12 ng of *p53* MO (*n*=77/85). (K) Side view of a 24 hpf embryo co-injected with 12 ng of *p53* MO and 8 ng of *rab5b* MO (*n*=98/108) (L) Side view of a 48 hpf embryo injected with 12 ng of *p53* MO. (M) Side view of a 48 hpf embryo co-injected with 12 ng of *p53* MO and 8 ng of *rab5b* MO (*n*=52/62). (N) Magnification of trunk region showing somites and notochord in 48 hpf control-injected embryos. (O) Magnification of trunk region showing somites and notochord in 48 hpf *rab5b* MO-injected embryos. (P) Magnification of trunk region showing somites in a 48 hpf control-injected embryo stained with phalloidin. (Q) Magnification of trunk region showing somites in a 48 hpf *rab5b* MO-injected embryo stained with phalloidin. (R) Magnification of trunk region showing somites and notochord in 48 hpf *p53* MO injected embryos. (S) Magnification of trunk region showing somites and notochord in 48 hpf *p53* MO and *rab5b* MO co-injected embryos. (T) Magnification of trunk region showing somites in a 48 hpf *p53* MO injected embryo stained with phalloidin. (U) Magnification of trunk region showing somites in a 48 hpf *p53* MO and *rab5b* MO co-injected embryo stained with phalloidin. (V) Lateral view of a 30 hpf embryo injected with 5 ng of control MO (*n*=92/95). (W) Lateral view of a 30 hpf embryo injected with 6 ng of rab5c MO (*n*=174/175). (X) Lateral view of a 48 hpf embryo injected with 5 ng of control MO (*n*=92/95). (Y) Lateral view of a 48 hpf embryo injected with 6 ng rab5c MO (*n*=158/159). (Z) Lateral view of a 30 hpf embryo injected with 9 ng of *p53* MO (*n*=54/54). (AA) Lateral view of a 30 hpf embryo co-injected with 9 ng of *p53* MO and 6 ng of *rab5c* MO (*n*=*n*=54/56). (AB) Lateral view of a 48 hpf embryo injected with 9 ng of *p53* MO. (AC) Lateral view of a 48 hpf co-injected with 9 ng of *p53* MO and 6 ng of *rab5c* MO (*n*=37/48). (AD) Magnification of trunk region showing somites and notochord in 48 hpf control-injected embryos. (AE) Magnification of trunk region showing somites and notochord in 48 hpf *rab5c* MO-injected embryos. (AF) Magnification of trunk region showing somites in a 48 hpf control-injected embryo stained with phalloidin. (AG) Magnification of trunk region showing somites in a 48 hpf *rab5c* MO-injected embryo stained with phalloidin. (AH) Magnification of trunk region showing somites and notochord in 48 hpf *p53* MO injected embryos. (AI) Magnification of trunk region showing somites and notochord in 48 hpf *p53* MO and *rab5c* MO co-injected embryos. (AJ) Magnification of trunk region showing somites in a 48 hpf *p53* MO injected embryo stained with phalloidin. (AK) Magnification of trunk region showing somites in a 48 hpf *p53* MO and *rab5c* MO co-injected embryo stained with phalloidin. (A, B, D, E are dorsal views, anterior to the left and the eyes were manually removed for simplification C is a side view, anterior to the left (‘ov’ indicates otic vesicle).

*rab5aa* MO-injected embryos (*n*=63/63) were morphologically indistinguishable from control-injected embryos (*n*=54/54). Due to a lack of obvious phenotype, we studied the expression pattern of *rab5aa* in detail. Before 24 hpf, *rab5aa* mRNA expression was found to be low and uniform throughout all tissues (data not shown). At 24 hpf, however, *rab5aa* mRNA was expressed in a subset of cells in the brain especially in the ventral anterior part of the neural tube, forebrain and midbrain region (Fig. 2(A)). Additionally, we found *rab5aa* mRNA expression in a bilateral patch of telencephalic cells (Fig. 2(B)). The hindbrain showed expression in the central region of each rhombomere (Fig. 2(C)), in cells in the outer region of the neural tube at the midbrain/hindbrain boundary (arrow in Fig. 2(D)) and at the posterior end of the hindbrain (arrows in Fig. 2(E)), which may correspond to expression in the anterior and posterior lateral line, respectively. Expression could also be seen in trunk region (Fig. 2(E)) showing positive cells scattered in the dorsal half of the embryo.

#### rab5b

At 24 hpf, *rab5b* MO-injected embryos showed thin and bar-shaped somites, as well as brain abnormalities. Specifically we found reduced forebrain and general brain necrosis (Fig. 2(G)), when compared to control-injected embryos (Fig. 2(F)). Double-injected *rab5b* MO / *p53* MO embryos showed the same curved axis and bar-shaped somites (Fig. 2(K)) as *rab5b* MO single-injected embryos (Fig. 2(G)) but with slightly fewer brain defects and less necrosis at 24 hpf. By 48 hpf *rab5b* MO-injected embryos showed a reduced and curved axis with curved notochord (Fig. 2(I)), more pronounced bar-shaped somites (Fig. 2(O)) and disrupted muscle fibres (Fig. 2(Q)) when compared to control-injected embryos (Fig. 2(H), (N) and (P)). When control and *rab5b* MO-injected embryos were co-injected with the *p53* MO, both control and *p53* MO injected embryos (Fig. 2(J)) appeared similar to control embryos (Fig. 2(F)). By 48 hpf, *rab5b* MO/*p53* MO double-injected embryos injected embryos showed similar reduced and curved axis with curved notochord (Fig. 2(M)) and pronounced bar-shaped somites (Fig. 2(S)) as *rab5b* MO-injected embryos (Fig. 2(I), (O) and (Q)). Additionally, *rab5b* MO / *p53* MO double-injected embryos showed reduced head size (Fig. 2(M)) and disrupted muscle fibres (Fig. 2(U)).

#### rab5c

Similar to *rab5b* MO-injected embryos, *rab5c* MO-injected embryos showed U-shaped somites, shortened axis, forebrain defects and brain necrosis, with the head region appearing poorly developed at 24 hpf (Fig. 2(W)) when compare to control-injected embryos (Fig. 2(V)). On the second day of development, *rab5c* MO-injected embryos continued to develop poorly, with reduced head size, shorter axis and curved tail (Fig. 2(Y)), when compared to control-injected embryos (Fig. 2(X)). Additionally, in *rab5c* MO-injected embryos muscle fibres failed to align as smoothly (Fig. 2(AG)) and notochord cells failed to form proper vacuoles (Fig 2AE) when compared to controls (Fig. 2(AD) and (AF)). When the control and *rab5c* MO-injected embryos were co-injected with the *p53* MO the control and *p53* MO injected embryos (Fig. 2(Z)) looked similar to control embryos (Fig. 2(V)). The *rab5c* MO and *p53* MO injected embryos showed the same curved axis, U-shaped somites, forebrain defects and brain necrosis (Fig. 2(AA)) as *rab5c* MO-injected embryos (Fig. 2(W)) at 24 hpf. By 48 hpf *rab5c* MO and *p53* MO injected embryos were more adversely affected with a more pronounced curved axis and poorly developed head region (Fig. 2(AC)) than *rab5b* MO-injected embryos (Fig. 2(Y)). Double-injected *rab5c* MO / *p53* MO embryos showed similar U-shaped somites (Fig. 2(AI)) and more disorganised muscle fibres (Fig. 2(AK)) than embryos injected with *rab5c* MO alone (Fig. 2(AE) and (AG))

#### rab5ab

**Fig. 3:**
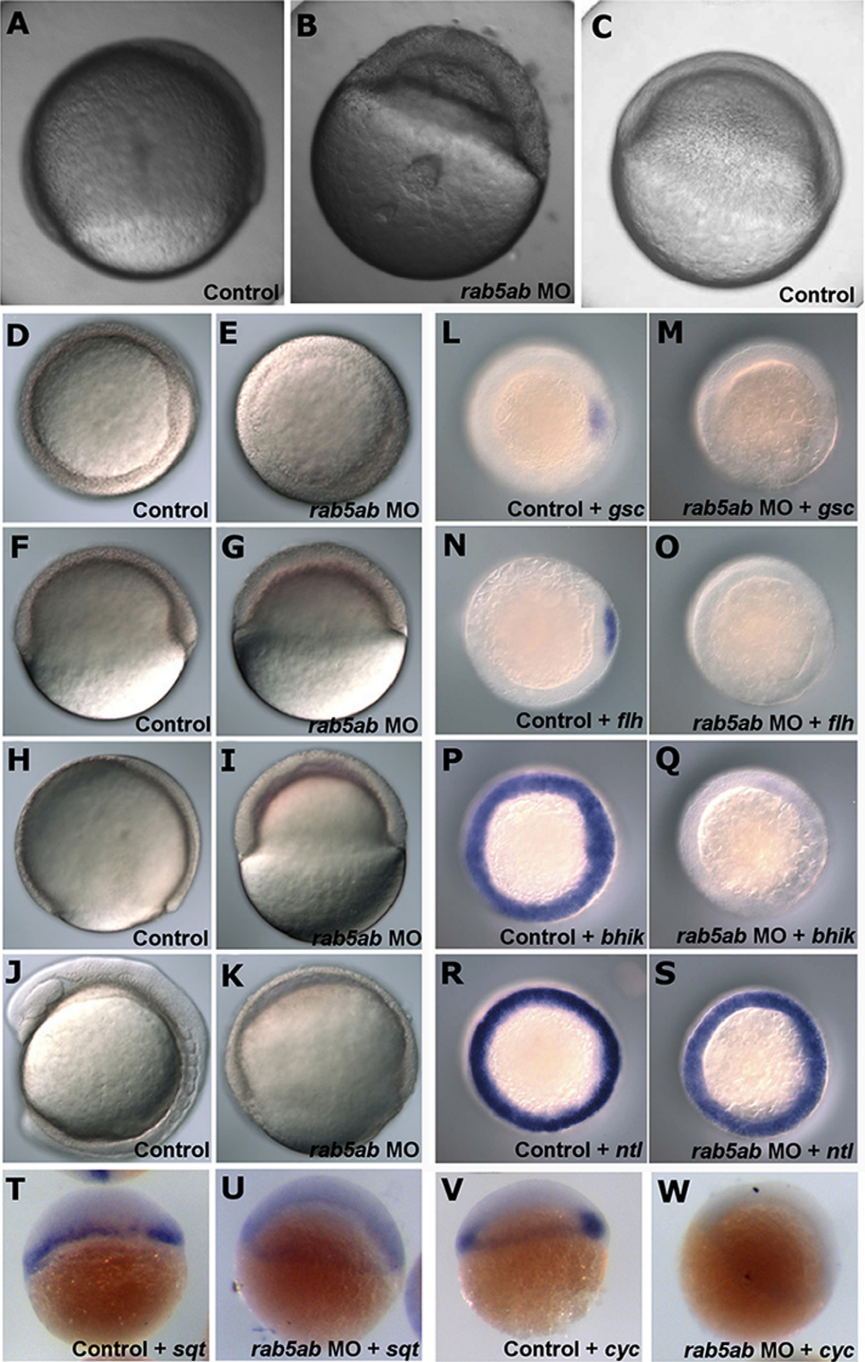
Loss of function of *rab5ab*. (A) Control embryo at 70% epiboly compared to (B) a 5 ng *rab5ab* MO-injected embryo at the same time point showing apparent accumulation of extracellular fluid between the yolk and the cells. (C) Control embryo at shield stage. (D) Animal view and (F) side view of a control-injected embryo at shield stage compared to (E) animal view and (G) side view of 3 ng rab5ab MO-injected embryos at the same time point. (H) Control-injected embryos at 90% epiboly compared to (I) the same time point in the 3 ng rab5ab MO-injected embryos. (J) 8 somite stage control embryo compared to (K) 3 ng rab5ab MO-injected embryo at the same time point. Expression pattern of *gsc* in (L) control MO-injected embryos (*n*=40/40) compared to (M) 3 ng *rab5ab* MO-injected embryos (*n*=41/41). Expression pattern of *flh* in (N) control MO-injected embryos (*n*=20/20) compared to (O) 3 ng *rab5ab* MO-injected embryos (*n*=21/21). Expression pattern of *bhik* in (P) control MO-injected embryos (*n*=29/29) compared to (Q) 3 ng *rab5ab* MO-injected embryos *n*=31/31). Expression pattern of *ntl* in (R) control MO-injected embryos (*n*=40/40) compared to (S) 3 ng *rab5ab* MO-injected embryos (*n*=39/39). Lateral view of expression pattern of *ndr1* in (T) control MO-injected embryos (*n*=30/30) compared to (U) *rab5ab* MO-injected embryos (*n*=30/30). Lateral view of expression pattern of *ndr2* in (V) control MO-injected embryos (*n*=30/30) compared to (W) *rab5ab* MO-injected embryos (*n*=29/29).

In contrast to *rab5aa*, knockdown of *rab5ab* produced a striking morphological phenotype during gastrulation. Specifically MO-injected embryos did not develop a dorsal organizer and died before the completion of epiboly (Fig. 3(B)). Development was dramatically slowed (Fig. 3(B)) in comparison with controls (Fig. 3(A)). Injection of 5 ng of *rab5ab* MO resulted in the embryos dying at between 30% and 50% epiboly. At 30% epiboly fluid had accumulated between blastoderm cells and the yolk cell (Fig. 3(B)). In these embryos, the cells of the blastoderm appeared substantially grainier in texture and less cohesive (Fig. 3(B)), when compared with the smooth blastoderm of the control-injected embryos (Fig. 3(A)) and stage matched control embryos (Fig. 3(C)). Further decreasing the dose of rab5ab MO to 3 ng (Fig. 3(E), (G), (I), (K)) resulted in *rab5ab* MO-injected embryos surviving to 80–90% epiboly (Fig. 3(K)). Embryos injected with *rab5ab* MO underwent a slowed epiboly (Fig. 3(E), (G), (I), (K)). Control embryos, however, underwent epiboly at a constant rate over approximately 5 h (Fig. 3(D), (F), (H), (J)). By the time control embryos reached 80% epiboly (Fig. 3(H)), MO-injected embryos had only progressed to 50% epiboly (Fig. 3(I)) and when MO-injected embryos eventually reached 80% epiboly (Fig. 3(K)), at 9 hpf, control embryos were at the 7-somite stage (Fig. 3(J)). Although many MO-injected embryos did not reach 80% epiboly, in cases where they did, the blastoderm margin contracted and pinched off the yolk, causing its contents to leak leading to the death of blastoderm cells. To confirm this phenotype we injected embryos with a second translation blocking MO to *rab5ab* (*rab5ab* MO2) and compared them with a 5 base-pair mismatch control MO (*rab5ab* MM MO2) and with uninjected control embryos. Once again the *rab5ab* MO injected embryos underwent a slowed epiboly, did not develop a dorsal organizer and died before the completion of epiboly (Fig S1H) when compared with both the *rab5ab* MM MO2 (Fig S1G) and the uninjected control (Fig S1F). The proportion of embryos that survive to 30% epiboly is significantly (*p*=0.002) reduced in embryos injected with *rab5ab* MO2 compared to those injected with *rab5ab* MM MO2 (Fig S1L)

**Fig. 4:**
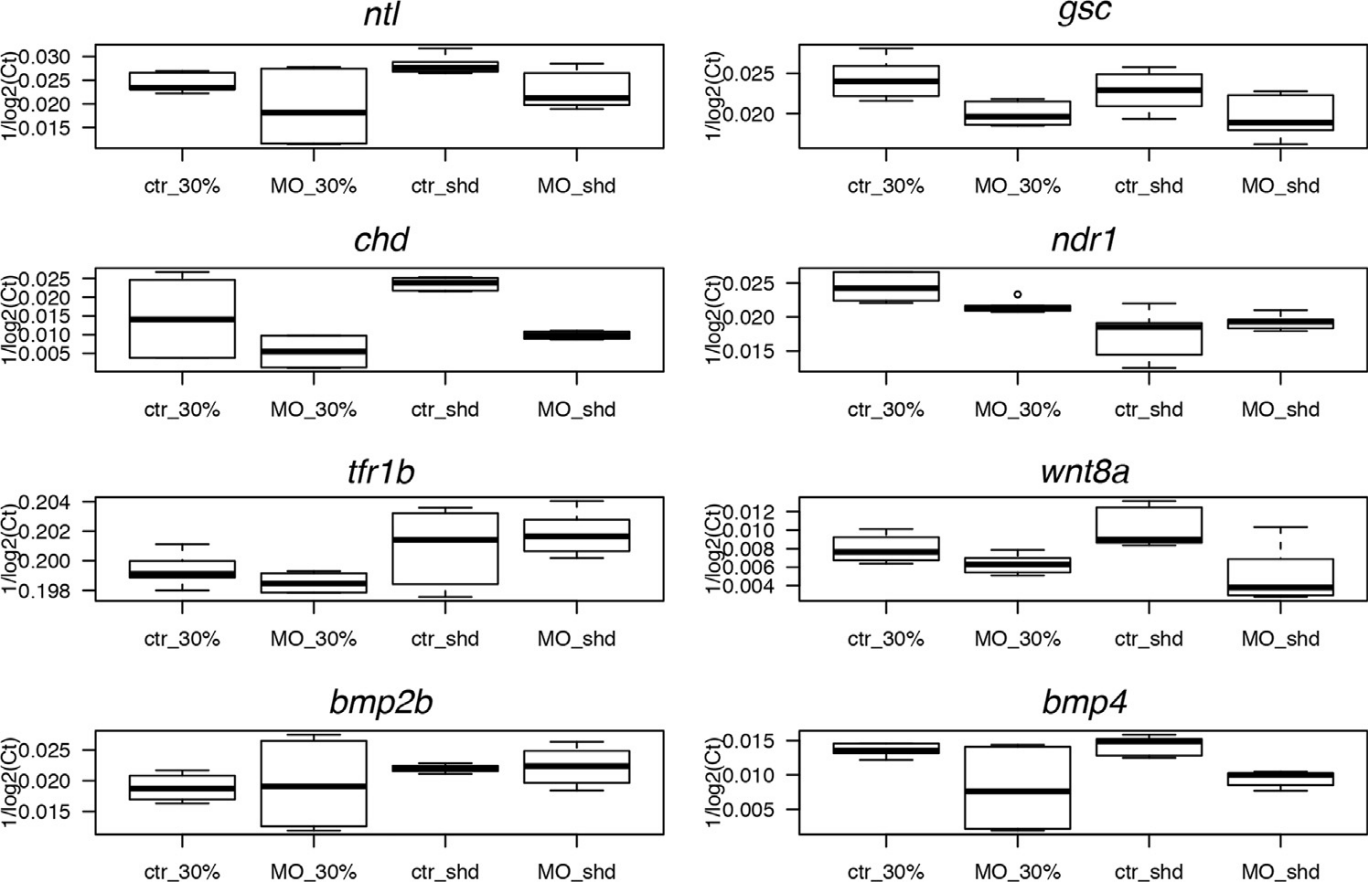
Quantitative analysis of morpholino activity. Expression of eight genes (*ntl*, *gsc*, *chd*, *ndr*1, *bmp2b*, *bmp4, wnt8a* and *tfr1b*) at two key developmental stages (30% epiboly and shield) was quantified using qRT-PCR, following MO or control injection. Ct data were 1/log_2_(Ct) transformed to stabilise variance and allow intuitive visual inspection of the data, *i.e.* a higher value equates to greater expression. Box-whisker plots show empirical distributions of gene expression for each stage X MO treatment. Horizontal lines denote median expression and boxes cover the interquartile range. Whiskers extend to 1.5 times the interquartile range, with additional outliers plotted as points.

The lack of visible dorsal organiser led us to investigate the mRNA components of the nodal signalling pathway. By *in situ* hybridisation we found that *rab5ab* MO-injected embryos showed no *gsc* (Fig. 3(M)), *flh* (Fig. 3(O)) or *bhik* (Fig. 3(Q)), expression compared to control MO-injected embryos (Fig. 3(L), (N), (P) respectively). There was some marginal expression of *ntl* (Fig. 3(S)) in *rab5ab* MO-injected embryos and reduced expression of *ndr1* (Fig. 3(U)) and *ndr2* (Fig. 3W) compared with controls (Fig. 3(R), (T), (V) respectively). Embryos injected with the second *rab5ab* MO also showed disruption of *gsc* expression (Fig S1K) while the 5-bp mismatch MO injected embryos (Fig S1J) and the uninjected control embryos showed normal expression (Fig S1I). To quantify and validate the *in situ* results we performed qRT-PCR on control and *rab5ab* MO injected embryos at 30% epiboly and shield stage. Empirical distributions of expression are shown in Fig. 4. For *gsc*, *chd*, *ntl* and *ndr1*, average expression was lower in MO-injected embryos, compared to control-injected embryos (*ntl*: Likelihood Ratio=12.4, *p*<0.001; *gsc*: L.R.=16.7, *p*<0.001; *chd*: L.R.=4.38, *p*=0.037), although this was only the case at 30% epiboly stage for *ndr*1 expression (morpholino×stage interaction: L.R.=15.6, *p*<0.001). For these measurements *transferrin receptor 1b (tfr1b)* was used as a control and showed no significant difference in expression between MO-injected and control-injected embryos (L.R.=0.097, *p*=0.76).

### A role for *rab5ab* in nodal signalling

To test whether the lack of nodal–responsive gene expression is specific to the *rab5ab* MO we compared expression of *gsc* in embryos co-injected with GFP-*rab5ab* RNA/*rab5ab* MO with those injected with a control MO or those injected with *rab5ab* MO alone. In embryos injected with GFP-*rab5ab* RNA/*rab5ab* MO (Fig S1C) and those injected with control MO (Fig S1A) we saw normal *gsc* expression. In those embryos injected with *rab5ab* MO alone (Fig S1B) we failed to see *gsc* expression.

To ensure the morpholino was specific we repeated the experiment using a second morpholino (*rab5ab* MO2) that binds to the UTR of *rab5ab*. Here we saw that the proportion of embryos that survived to 30% epiboly was significantly increased (*p*=0.004) in embryos co-injected with *rab5ab* RNA/*rab5ab* MO2 when compared with those injected with *rab5ab* MO2 alone (Fig S1L). *gsc* expression was also normal in embryos co-injection with *rab5ab* RNA/*rab5ab* MO2 (Fig S1Q) when compared with uninjected embryos (Fig S1M) and those injected with *rab5ab* MM MO2 (Fig S1N). *gsc* expression was missing from those embryos injected with *rab5ab* MO2 alone (Fig S1P). In addition, when downstream Nodal signalling was rescued by injection of 25 pg of activated *taram-a* RNA (Aoki et al., 2002; Aquilina-Beck et al., 2007), *gsc* expression was seen in both *rab5ab* MO-injected (Fig S1E) and control embryos (Fig S1D).

The effect of *rab5ab* on nodal signalling is likely due to maternal transcripts, as embryos injected with 10 ng of *rab5a2* splice MO were comparable to controls at shield stage showing a visible organizer unlike the *rab5a2* morpholino injected embryos. The *rab5a2* splice MO injected embryos also completed epiboly however by 24 hpf the *rab5a2* splice MO injected embryos showed an accumulation of dead cells across the yolk (Fig S1S) compared to control injected embryos (Fig S1R). Although the *rab5a2* splice MO injected embryos had massive cell death by 24 hpf, at shield stage they had a visible organizer, and expression patterns for *bhik*, *gsc*, *ntl* and *chd* were similar to controls (Fig S1T-AA)

**Fig. 5:**
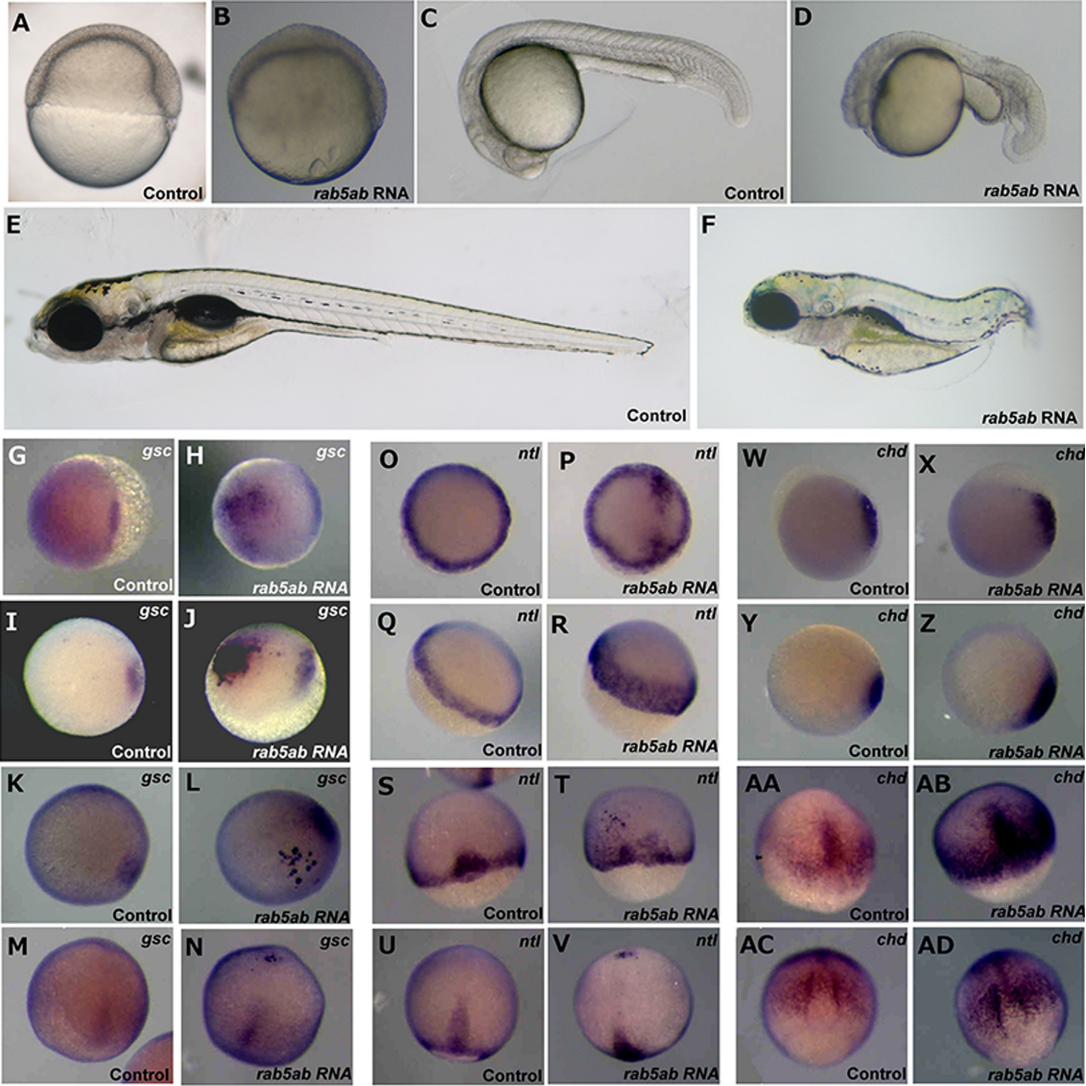
A role for *rab5ab* in Nodal signalling. Lateral view of (A) shield stage control injected embryo compared to (B) shield stage 1.5 ng *rab5ab* mRNA overexpressing embryo (*n*=14/41). Lateral view of (C) 24 hpf control injected embryo compared to (D) 24 hpf 1.5 ng *rab5ab* mRNA overexpressing embryo (*n*=12/39). Lateral view of (E) 5dpf control injected embryo compared to (F) 5dpf 1.5 ng *rab5ab* mRNA overexpressing embryo (*n*=38/38). Expression of *gsc* in control embryos at (G) 30%, (I) 50%, (K) 70% and (M) 90% epiboly compared to expression of *gsc* in *rab5ab* overexpressing embryos at (H) 30% (*n*=10/12), (J) 50% (*n*=20/21), (L) 70% (*n*=8/13) and (N) 90% epiboly (*n*=5/10). Expression of *ntl* in control embryos at (O) 30%, (Q) 50%, (S) 70% and (U) 90% epiboly compared to expression of *ntl* in *rab5ab* overexpressing embryos at (P) 30% (*n*=8/12), (R) 50% (*n*=21/22), (T) 70% (*n*=9/13) and (V) 90% epiboly (*n*=7/10). Expression of *chd* in control embryos at (W) 30%, (Y) 50%, (AA) 70% and (AC) 90% epiboly compared to expression of *ntl* in *rab5ab* overexpressing embryos at (X) 30% (*n*=12/12), (Z) 50% (*n*=11/11), (AB) 70% (*n*=10/10) and (AD) 90% epiboly (*n*=10/10). The *gsc* expression patterns are shown as animal pole views as are 30% epiboly *ntl* expressing embryos and 30% and 50% *chd* expressing embryos. The remainder of the embryos are shown as a side view for improved visualisation of expression patterns.

Wild-type *rab5ab* was overexpressed in normal embryos by injecting 1.5 ng of synthetic 5′-capped RNA. At 40–50% epiboly, an accumulation of cells was seen on the animal pole of approximately one third of the *rab5ab* RNA-injected embryos (*n*=14/41) (Fig. 5(B)). In the remaining *rab5ab* RNA-injected embryos, the embryonic shield appeared larger (*n*=27/41). At 24 hpf, approximately two thirds of the *rab5ab* RNA-injected embryos appeared similar to control embryos, except for an enlarged yolk extension (*n*=27/39). The remaining third displayed a reduced body axis and reduced head size (*n*=12/39) (Fig. 5(D)). By 5 dpf, all of the *rab5ab* RNA-injected embryos showed a severely shortened body axis and thicker, less extended yolks (*n*=38/38) (Fig. 5F).

To establish whether overexpression of *rab5ab* affected nodal-responsive genes, we examined expression of the dorsal markers *chd*, *gsc* and *ntl*. At 30% epiboly, *rab5ab* RNA-injected embryos showed expression of *gsc* in the ventral region, in addition to the normal dorsal expression (Fig. 5(H)). Additionally, some *rab5ab* RNA-injected embryos expressed *ntl* in patches in the animal pole (Fig. 5(P)) in addition to the normal marginal expression. At 50% epiboly, *rab5ab* RNA-injected embryos showed ectopic *gsc* expression in the animal pole (Fig. 5(J)). At this stage, the embryos showed no ectopic *ntl* expression. However, *rab5ab* RNA-injected embryos did show abnormal *ntl* expression, which was expanded toward the animal pole from the normal marginal expression domain (Fig. 5(R)). At 70% epiboly, *rab5ab* RNA-injected embryos showed additional *gsc* expression in the animal pole (Fig. 5(L)). Similarly *ntl* in *rab5ab* RNA-injected embryo was ectopically expressed at the animal pole (Fig. 5(T)). At 90% epiboly *rab5ab* RNA-injected embryos continued to show mislocalised expression of both *gsc* (Fig. 5(N)) and *ntl* (Fig. 5(V)). Expression of *chd* was unchanged in experimental embryos, compared to the control injected embryos in 30% (Fig. 5(X)), 50% (Fig. 5(Z)), 70% (Fig. 5(AB)) and 90% epiboly (Fig. 5(AD)).

### Further studies of *rab5ab* function

**Fig. 6:**
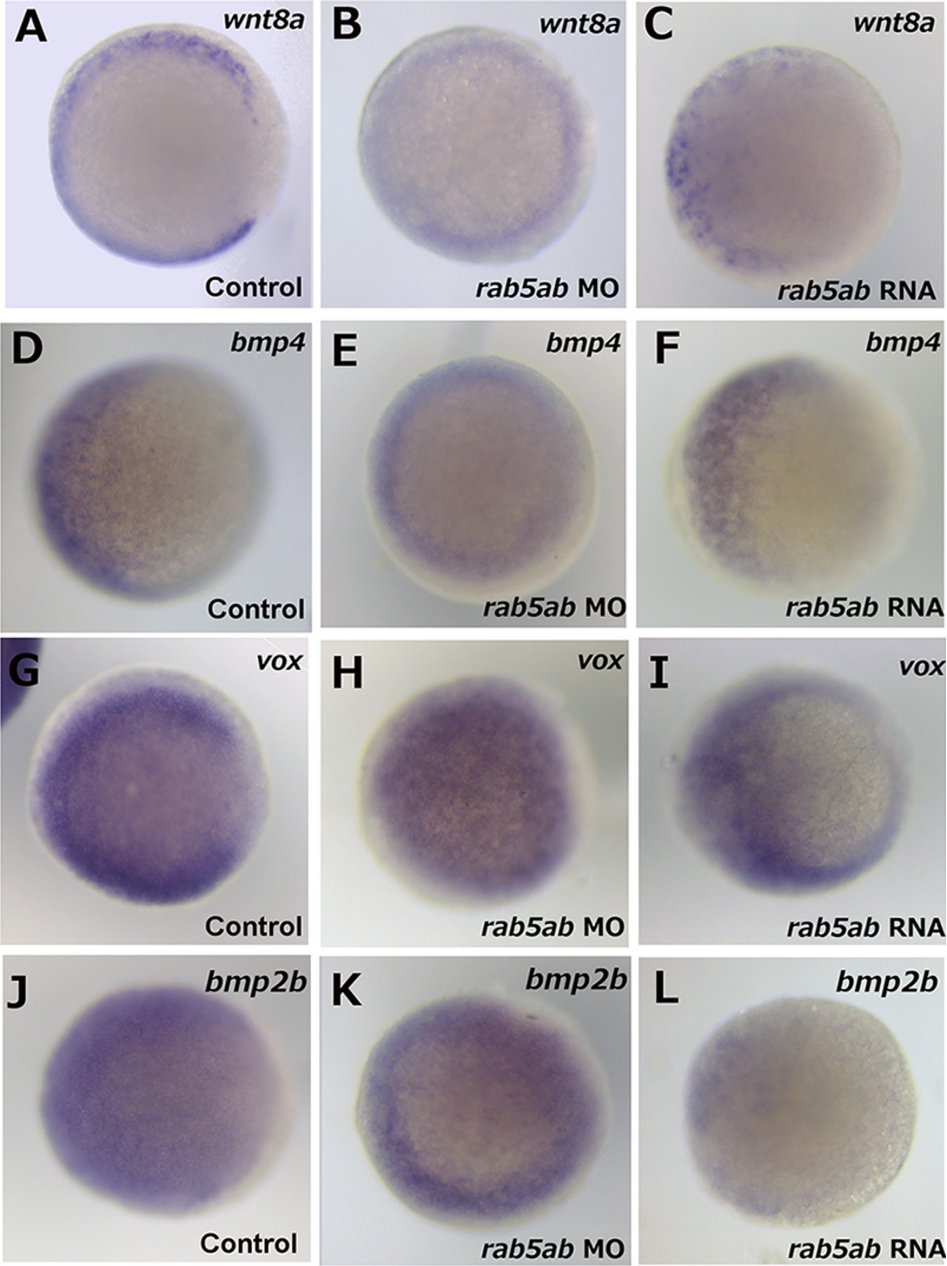
The role of *rab5ab* in ventral gene expression. Expression of *wnt8a* in (A) control injected embryos (*n*=27/27), (B) *rab5ab* MO injected embryos (*n*=29/29) and (C) *rab5ab* RNA injected embryos (*n*=41/42) at 30% epiboly. Expression of *bmp4* in (D) control injected embryos (*n*=17/17), (E) *rab5ab* MO injected embryos (*n*=27/27) and (F) *rab5ab* RNA injected embryos (*n*=20/20) at 30% epiboly. Expression of *vox* in (G) control injected embryos (*n*=47/47), (H) *rab5ab* MO injected embryos (*n*=32/32) and (I) *rab5ab* RNA injected embryos (*n*=55/55) at 30% epiboly. Expression of *bmp2b* in (J) control injected embryos (*n*=41/41), (K) *rab5ab* MO injected embryos (*n*=36/36) and (L) *rab5ab* RNA injected embryos (*n*=41/41) at 30% epiboly. All embryos shown are animal view.

As injection of *rab5ab* RNA resulted in an unexpected pattern of expression of nodal downstream genes *ntl* and *gsc* while injection of *rab5ab* MO resulted in abolishment of these genes and qRT-PCR showed a reduction in *chd* and *ndr1* expression, we investigated the role of *rab5ab* in the expression of ventral markers *wnt8a, bmp4, vox* and *bmp2b*. Injection of *rab5ab* MO resulted in widespread expression of *wnt8a* around the whole margin (Fig. 6(B)) compared to control embryos where expression was excluded from the dorsal margin (Fig. 6(A)). Measurement of mRNA expression using qRT-PCR showed the level of *wnt8a* to be lower (L.R.=32.0, *p*<0.001) in *rab5ab* MO injected embryos compared to controls (Fig. 4). Injection of *rab5ab* RNA resulted in the expression of *wnt8a* being restricted to the ventral most half of the embryo (Fig. 6(C)).

*In situ* hybridisation showed that *bmp4* expression in embryos injected with *rab5ab* MO was primarily in the margin (Fig. 6(E)) compared to control embryos where *bmp4* expression could be observed over the ventral half of the embryos (Fig. 6(D)). Similarly, qRT-PCR showed the level of *bmp4* to be lower (L.R.=23.4, *p*<0.001) in *rab5ab* MO injected embryos compared to controls (Fig. 4). Injection of *rab5ab* RNA resulted in the expression of *bmp4* being further restricted to the ventral most part of the embryo (Fig. 6(F)) when compared with controls (Fig. 6(D)). Expression of *vox* in embryos injected with *rab5ab* MO was observed over the whole animal pole of the embryo (Fig. 6(H)) compared to controls where the expression was excluded from the dorsal most part of the embryo (Fig. 6(G)) in embryos injected with *rab5ab* RNA, *vox* expression was excluded from the majority of the dorsal half of the embryo (Fig. 6(I)).

Expression of *bmp2b* in embryos injected with *rab5ab* MO was observed predominantly in in the margin of the embryo (Fig. 6(K)) compared to controls where the expression was excluded from the dorsal most part of the embryo only (Fig. 6(J)). Measurement of *bmp2b* levels by qRT-PCR showed no significant difference (L.R.=0.267, *p*=0.61) between embryos injected with *rab5ab* MO and controls (Fig. 4). In embryos injected with *rab5ab* RNA *bmp2b* expression was excluded from the majority of the dorsal half of the embryo (Fig. 6(L)).

### Control of epiboly movements by Rab5ab

**Fig. 7:**
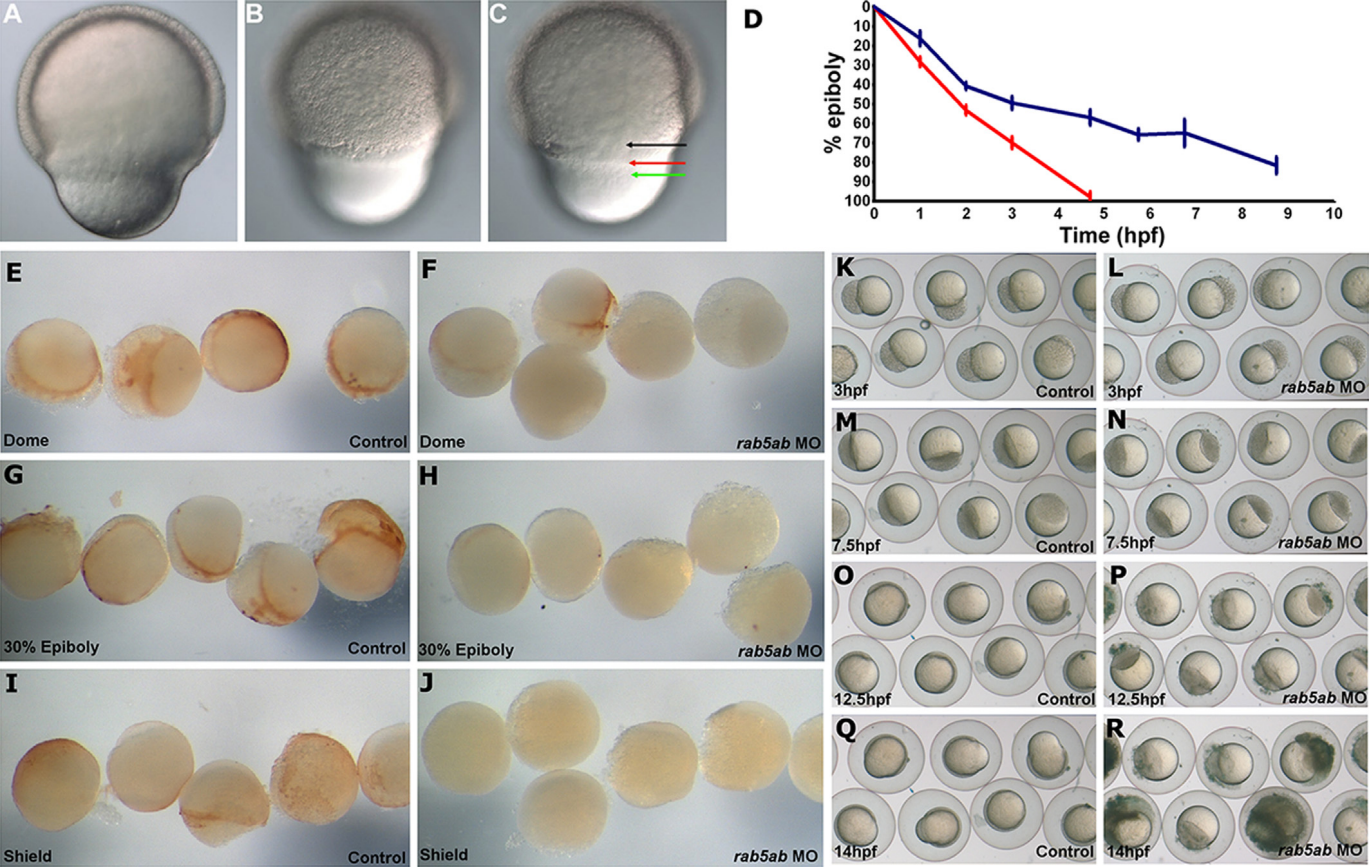
Roles for *rab5ab* in endocytosis and epiboly. (A), (B) and (C) Lateral views, (dorsal to the right) of epiboly of cells of the blastoderm (black arrow), enveloping layer (red arrow) and yolk syncytial layer (green arrow) in a 70% epiboly stage 3 ng *rab5ab* MO injected embryo in three different focal planes. (D) Graph shows the progression of epiboly in rab5ab MO-injected embryos (blue line) and in control embryos (red line) (*n*=12). Animal view of control embryos at (E) dome (G) 30% epiboly and (I) shield stage compared to (F) dome (*n*=13/17), (H) 30% epiboly (*n*=12/13) and (J) shield stage (*n*=14/15) in 5 ng *rab5ab* MO injected embryos. The brown staining shows the uptake of biotin via endocytosis during epiboly. Control embryos (*n*=16/16) subjected to cold shock at (K) 3 hpf, (M) 7.5 hpf, (O) 12.5 hpf and (Q) 14 hpf when compared to *rab5ab* MO-injected embryos (*n*=14/14) subjected to cold shock at (L) 3 hpf, (N) 7.5 hpf, (P) 12.5 hpf and (R) 14 hpf.

In *rab5ab* MO-injected embryos the movement of all tissues layers was significantly delayed. Embryos injected with *rab5ab* MO underwent a slowed epiboly from the outset and slowed further as epiboly progressed, whereas controls underwent epiboly at a constant rate over approximately 5 h (Fig. 7(D)). This delay was initially synchronous but in embryos that survived through later stages of epiboly, we found that the delayed movement of distinct layers was out of sync (Fig. 7(A), (B) and (C)). Since epiboly in zebrafish involves endocytic removal of the yolk cell membrane in the cells (Betchaku and Trinkaus, 1978; Solnica-Krezel and Driever, 1994; Lepage et al., 2014) we investigated endocytosis in the *rab5ab* MO-injected embryos. To measure the endocytosis activity directly we incubated *rab5ab* MO-injected embryos and control-MO injected embryos in a physiological solution containing biotinylated dextran, then fixed the embryos at three stages. Control embryos, at dome stage and 30% epiboly, all showed a ring of staining for biotinylated dextran around the leading edge of the blastoderm (Fig. 7(E) and (G)). At shield stage, this staining formed a gradient from the dorsal to ventral side of the embryo (Fig. 7(I)). In contrast, *rab5ab* MO-injected embryos showed very little staining at dome stage, less staining at 30%, and no staining at shield stage (Fig. 7(F), (H) and (J)).

Despite the defects in endocytosis in the *rab5ab* MO-injected embryos, epiboly did proceed but at a slower pace and did not finish. This suggested that the microtubules in the yolk were unaffected and were responsible for epiboly proceeding as far as it did. As cold depolymerizes microtubules (Jesuthasan and Stahle, 1997), we held 5 ng *rab5ab* MO-injected and control embryos at 20 °C and monitored for 18 h (Mov S1). Control embryos developed normally (Fig. 7(K), (M), (O) and (Q)) albeit with some developmental delay, whereas *rab5ab* MO-injected embryos arrested and started to die at 13 hpf (10 h in to monitoring) at sphere to early epiboly stages (Fig. 7(L), (N), (P) and (R)). *rab5ab* MO-injected siblings incubated at 28 °C died at the later stage of 70% epiboly, while control-MO injected siblings incubated at 28 °C developed normally.

Supplementary material related to this article can be found online at doi:10.1016/j.ydbio.2014.11.007.

The following is the Supplementary material related to this article Movie S1.

Movie S1 Time-lapse video comparing the development of embryos held at 20 °C injected with either control MO or *rab5ab* MO. The upper half of the frame shows the development of control MO injected embryos while the lower half shows *rab5ab* MO injected embryos. The video starts with embryos at 3 hpf and images are taken for 18 h.

### Activity of *rab5ab* in endocytosis

**Fig. 8:**
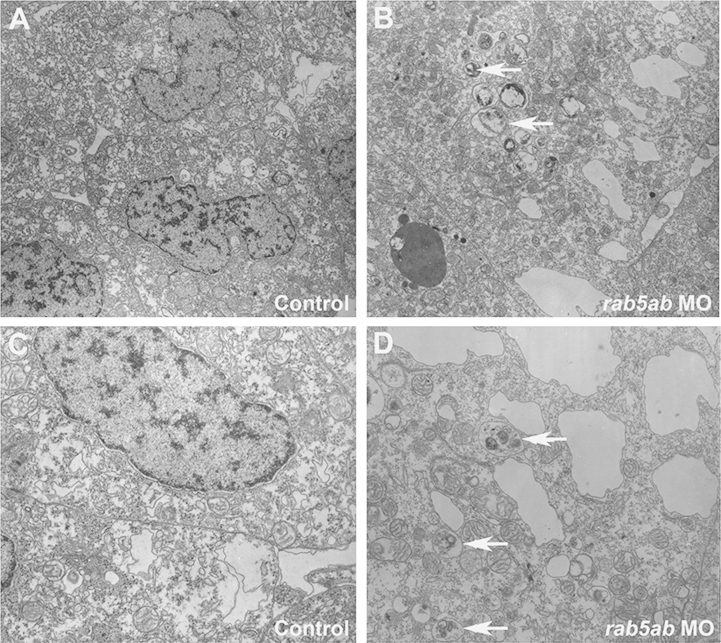
Activity of *rab5ab* in endocytosis. Transverse sections of cells of the leading edge of the enveloping layer from a 3 ng control-injected embryo 80% epiboly (A) and (C), a 3 ng *rab5ab* MO injected embryo (fixed at 40% epiboly but when control embryos were at 80% (B) and (D). White arrows show large secondary lysosomes with membranous contents. (A) and (B) are at 10,000×; and (C) and (D) are at 18,750×.

The mammalian version of RAB5A has been shown to function as a regulatory factor in the early endocytosis pathway by stimulating membrane fusion during endocytosis (Gorvel et al., 1991; Bucci et al., 1992; Stenmark et al., 1994). We investigated the cell morphology of *rab5ab* MO-injected cells at the ultrastructural level (Fig. 8) and found that *rab5ab* MO-injected cells showed enlarged, smooth membrane profiles with highly irregular shapes (Fig. 8(B) and (D)), which were not observed in the cells of control-injected embryos (Fig. 8(A) and (C)). Additionally, the cells of *rab5ab* MO-injected embryos had an increased number of what appear to be large secondary lysosomes (Fig. 8(B) and (D) white arrows).

## Discussion

The RAB5 family has been one of the most extensively studied of the Rabs (Li and Stahl, 1993; Singer-Kruger et al., 1994; Zerial and McBride, 2001). Their role in vesicle trafficking and endocytosis within the cell is well characterised (Gorvel et al., 1991; Bucci et al., 1992) and, for this reason, the Rab5 family has been used to understand signalling in the developing embryo (Scholpp and Brand, 2004; Hagemann et al., 2009). The transport of signalling factors in and out of a cell is integral to the patterning of the embryo, with the Rab5 family being used to understand the endocytosis that control this signalling. However, comparative studies between the genes within the *rab5* family have not been undertaken in whole animal systems previously and the possibility that the various *rab5* genes perform different developmental roles is hitherto unexplored. We sought to distinguish the possibility that *rab5* genes are functionally redundant with overlapping activities, from the possibility that *rab5* genes have divergent functions during development.

It was important first to account for all of the *rab5* gene family members in zebrafish and we found that there are four rab5 family members: *rab5aa*, *rab5ab*, *rab5b* and *rab5c*, which compares with the three *rab5* genes in human. The duplicated rab5a gene is particularly interesting, since the knockdown of each of these two genes resulted in very different phenotypes in our study, whereas *rab5b* and *rab5c* showed similar phenotypes. Specifically, *rab5aa* MO-injected embryos were phenotypically indistinguishable from controls, while rab5ab MO-injected embryos showed an early lethal phenotype.

The lack of an abnormal phenotype associated with *rab5aa* knockdown suggests that it is redundant for early development, although it may be required for post-embryonic development. Indeed, expression data suggests a subtle role for *rab5aa* in later brain development (Fig. 2) with other animal models showing a role for Rab5a in the brain (Allende and Weinberg, 1994; de Hoop et al., 1994; Sadler et al., 2007; Rosenegger et al., 2010). In rats, Rab5a has been detected in axons and dendrites, with Rab5a co-localised with synaptophysin-containing vesicles, suggesting a role for Rab5a in axonal and dendritic endocytosis (De Hoop et al., 1994). Our results show expression of *rab5aa* in zebrafish is restricted to discrete parts of the brain and spinal cord.

We found that knockdown of *rab5b* and *rab5c* lead to similar abnormal phenotypes. Specifically, MO-injected embryos showed no obviously abnormal phenotype through gastrulation but by 24 hpf had thin and bar-shaped somites, forebrain defects and cell death, suggesting a later role for these *rab5* genes (Fig. 2). *In situ* hybridisation shows *rab5b* expression from 1–13 somites in the YSL and pronephric mesoderm, then after 20 somites in the YSL, pronephric ducts and dorsal telencephalon (Thisse and Thisse, 2004). Taken together, these data imply a role for rab5b in nervous system development. In rat hippocampal cultures, Rab5b has been shown to be upregulated by the neuroprotective agent DHPG (Blaabjerg et al., 2003). Neuroprotection by DHPG against NMDA-mediated injury may involve facilitation of NMDA receptor endocytosis stimulated by a DHPG-induced increase in Rab5b synthesis and may therefore play a role in synaptic plasticity (Arnett et al., 2004; Baskys et al., 2007).

Knockdown of *rab5c*, although phenotypically similar to knockdown of *rab5b*, suggests that *rab5c* and *rab5b* may have different roles in development. *In situ* hybridisation data for *rab5c* showed expression from the 20 somites to the Prim-15 stage in the intermediate cell mass of mesoderm, the site of primitive hematopoiesis (Detrich et al., 1995; Thisse and Thisse, 2004). At later stages of development, *rab5c* expression is seen in axial vasculature and blood (Thisse and Thisse, 2004). A previous study of knock down of *rab5c* reported cell death over the whole embryo resulting in reduced yolk extension and expanded hindbrain at 28 hpf and at 56 hpf there was significant cell death, resulting in small head and eyes and bending of the body axis (Kalen et al., 2009). This result corresponds well with what we have observed, which included shortened axis and brain cell death. Taken together these observations suggest a distinct function for *rab5c* in mesoderm development at the late gastrula stage as it has been observed that Wnt11 functions in gastrulation by controlling cell cohesion through Rab5c and E-cadherin (Ulrich et al., 2005).

Depletion of *rab5ab* led to loss of the dorsal organiser and embryonic lethality by 90% epiboly stage. We therefore examined the expression of the nodal genes *ndr1* and *ndr2* and their downstream genes *gsc*, *flh* and *ntl* and found all but *ntl* to be abolished in rab5ab MO-injected embryos. Further this loss of expression for *gsc*, could be reversed by injection of synthetic *rab5ab* or *taram-a* mRNA. Indeed injection of large amounts of *rab5ab* RNA resulted in ectopic expression of downstream genes *gsc* and *ntl* but not the dorsal marker gene *chd*. It also resulted in embryos with larger organizers and shorter body axes. Interestingly *ntl* showed no ectopic expression at shied stage but instead showed expansion of the margin into the animal pole. This and the presence of *ntl* in rab5ab MO injected embryos could be explained by the fact that the expression of *ntl* is not entirely nodal-related, but is also regulated by Wnt and BMP signalling (Harvey et al., 2010). We therefore investigated whether *bmp* and *wnt* signalling might be affected in embryos overexpressing *rab5ab* or in those injected with a rab5ab morpholino. Although *wnt8a* was present around the whole margin of the embryo total *wnt8a* expression was lower in embryos injected with rab5ab MO. In embryos injected with *rab5ab* RNA expression of *wnt8a* was restricted to the ventral half of the embryo. Expression of *bmp* family members was more complex and while *bmp4* expression was decreased in *rab5ab* MO injected embryos, there was no significant change in *bmp2b* expression in these embryos. Expression of *bmp4*, *bmp2b* and *vox* showed abnormal distribution both in embryos overexpressing *rab5ab* and those injected with *rab5ab* MO. It is possible that *bmp2b* may be driving *ntl* expression in those embryos lacking *rab5ab*. All together this shows an important role for *rab5ab* in nodal signalling and dorsal-ventral patterning.

In addition to its role in Nodal signalling we find *rab5ab* plays a role in cell movement within the developing embryo. In *rab5ab* MO-injected embryos, epiboly is slowed. This is understandable, as RAB5 family members are known for their role in endocytosis (Bucci et al., 1995). Epiboly is thought to be the result of two processes, endocytosis at the margin, which moves cells over the yolk, and the contraction of the actin cytoskeleton within the yolk cell proper (Solnica-Krezel and Driever, 1994). Therefore cell movements within the developing embryo are disrupted when endocytosis associated with epiboly is diminished (Fig. 6) (Lepage et al., 2014) but when we further disrupt the microtubule cytoskeleton in the yolk, epiboly is not rescued. Moreover, it appears that certain events such as closing of the actin ring at the end of epiboly are independent of the epiboly process as this occurs whether epiboly completes or not. It is also possible that the start of gastrulation may be independent of the stage of epiboly, as marginal expression of *bmp4* and *bmp2b* is seen in embryos injected with rab5ab MO at 30% epiboly where as in control embryos this is not be observed until the embryos enter gastrulation at 50% epiboly.

Non-embryonic *nodal* transcripts in the YSL can mediate interaction between the embryonic and non-embryonic tissues that maintain *nodal* related gene expression in the margin (Fan et al., 2007). Additionally *ndr1* function is required in the YSL to induce the morphological shield, and the YSL is a source of Nodal signals that is independent of the population in the overlying blastomeres. Both Nodal ligands Ndr1 and Ndr2 are expressed by the YSL and induce *ndr1* mRNA expression in the overlying blastomeres. It has been suggested that the three non-embryonic sources of Nodal ligands, maternal *ndr1* and non-embryonic *ndr1* and *ndr2*, account for the complete spectrum of early nodal signalling and, therefore, organizer specification and induction of mesoderm and endoderm (Hong et al., 2011). A recent paper (Kumari et al., 2013) has shown that maternal control of Nodal signalling is via the conserved Y box-binding protein 1 (ybx1) and that maternal-effect mutations in zebrafish *ybx1* lead to deregulated Nodal signalling, gastrulation failure, and embryonic lethality. The paper suggests that Ybx1 prevents ectopic Nodal activity.

Our data and the published literature lead us to propose that Nodal signals emanating from the YSL are taken up by blastomeres via endocytic vesicles under the control of Rab5ab. This model could explain the abnormal accumulation of fluid we observe between the blastoderm and the yolk in rab5ab MO-injected embryos (Fig. 3(B)). In addition, *rab5ab* over–expressing embryos showed ectopic expression, as well as normal expression of the markers of Nodal signalling *gsc* and *ntl*. This would be consistent with the model if early expression of these genes was controlled by maternal and/or non-embryonic sources of Nodal ligands but later expression was due to embryonic sources. An alternative scenario is that that maternal *rab5ab* is somehow involved in the *ybx1* maternal control of Nodal signalling leading to deregulation of nodal signalling, its downstream genes and deregulation of DV patterning.

It was recently shown that zebrafish dynamin, a GTPase required for receptor-mediated endocytosis, plays a fundamental role within the blastoderm during epiboly. Dynamin is required for completion of epiboly and maintains epithelial integrity and the transmission of tension across the EVL (Lepage et al., 2014). Embryos lacking dynamin show a similar phenotype to those we have shown lacking *rab5ab*. In *Drosophila*, remodelling of the apical surface during epithelial morphogenesis has been shown to be regulated by dynamin and the Rab5-effector Rabankyrin-5 (Fabrowski et al., 2013) while research in the sea urchin embryo suggests that dynamin-mediated endocytosis acts as a sink to limit the range of Nodal signalling (Ertl et al., 2011). In sea urchins, inhibition of dynamin, resulted in embryos that became radialised and phenocopied embryos that overexpress *nodal.* Although this does not correspond with what we see with knock down of *rab5ab* it does suggest a possible relationship between rab5ab, *dynamin* and *nodal* which is worthy of further study.

In conclusion, the key finding of this study is the crucial role for Rab5ab in early nodal signalling and organizer specification in the developing zebrafish embryo. It should also be noted that various members of the Rab5 family are associated with different roles in early embryonic development. Corroborative evidence from whole organism phenotypic analysis in zebrafish is more consistent with functional divergence, than redundancy, between *rab5* genes.

## Appendix A Supplementary materials

Supplementary data Supplemental Fig. 1. Rescue of *rab5ab* MO׳s and phenotype of *rab5ab* splice MO. Lateral view of *gsc* expression in (A) control MO-injected embryos (*n*=30/30) compared to (B) *rab5ab* MO-injected embryos (*n*=30/30) and (C) *rab5ab* MO and *rab5ab* RNA injected embryos (*n*=30/31). Lateral view of *gsc* expression in (D) control and *Taram-A* RNA injected embryos (*n*=20/20) and (E) *rab5ab* morpholino and Taram-A RNA-injected embryos (*n*=20/20). Lateral view of (F) control uninjected embryos (*n*=62) (G) *rab5ab* mismatch MO2-injected embryos (*n*=119/119) and (H) *rab5ab* MO2 injected embryos when compared at the same time point (*n*=102/103). Animal view of *gsc* expression in 50% epiboly stage (I) control uninjected embryos (*n*=10/10) compared to (J) *rab5ab* mismatch (MM) MO2-injected embryos (*n*=10/10) and (K) *rab5ab* MO2 injected embryos (*n*=10/11). (L) Box-whisker plots show empirical distributions of uninjected, *rab5ab* MM MO2 injected, *rab5ab* MM MO2+RNA injected, *rab5ab* MO2 and *rab5ab* MO2+RNA injected embryos that survive to 30% epiboly. Horizontal lines denote median expression and boxes cover the interquartile range. Whiskers extend to 1.5 times the interquartile range, with additional outliers plotted as points. Animal view of *gsc* expression in 50% epiboly stage (M) control uninjected embryos (*n*=31/31) compared to (N) *rab5ab* MM MO2 injected embryos (*n*=51/51), (O) *rab5ab* MM MO2+RNA injected embryos, (P) *rab5ab* MO2 injected embryos (*n*=20/33) and (Q) *rab5ab* MO2+RNA injected embryos (*n*=36/42). Side view of (R) 24 hpf control embryo (*n*=30), side view of (S) embryos injected with 10 ng of *rab5ab* splice MO (*n*=30). Animal view of expression pattern of *bhik* in control (T) and *rab5ab* splice (U) morpholino injected embryos. Animal view of expression pattern of *gsc* in control (V) and *rab5ab* splice (W) morpholino injected embryos. Animal view of expression pattern of *ntl* in control (X) and *rab5ab* splice (Y) morpholino injected embryos. Animal view of expression pattern of *chd* in control (Z) and *rab5ab* splice (AA) morpholino injected embryos (*n*=10/12).
