# Supplementary figures and images for: Zebrafish Rab5 proteins and a role for Rab5ab in nodal signalling
Source: Dev Biol. 2015 Jan 15;397(2):212–24. doi: 10.1016/j.ydbio.2014.11.007 (PMC4294769; doi:10.1016/j.ydbio.2014.11.007)

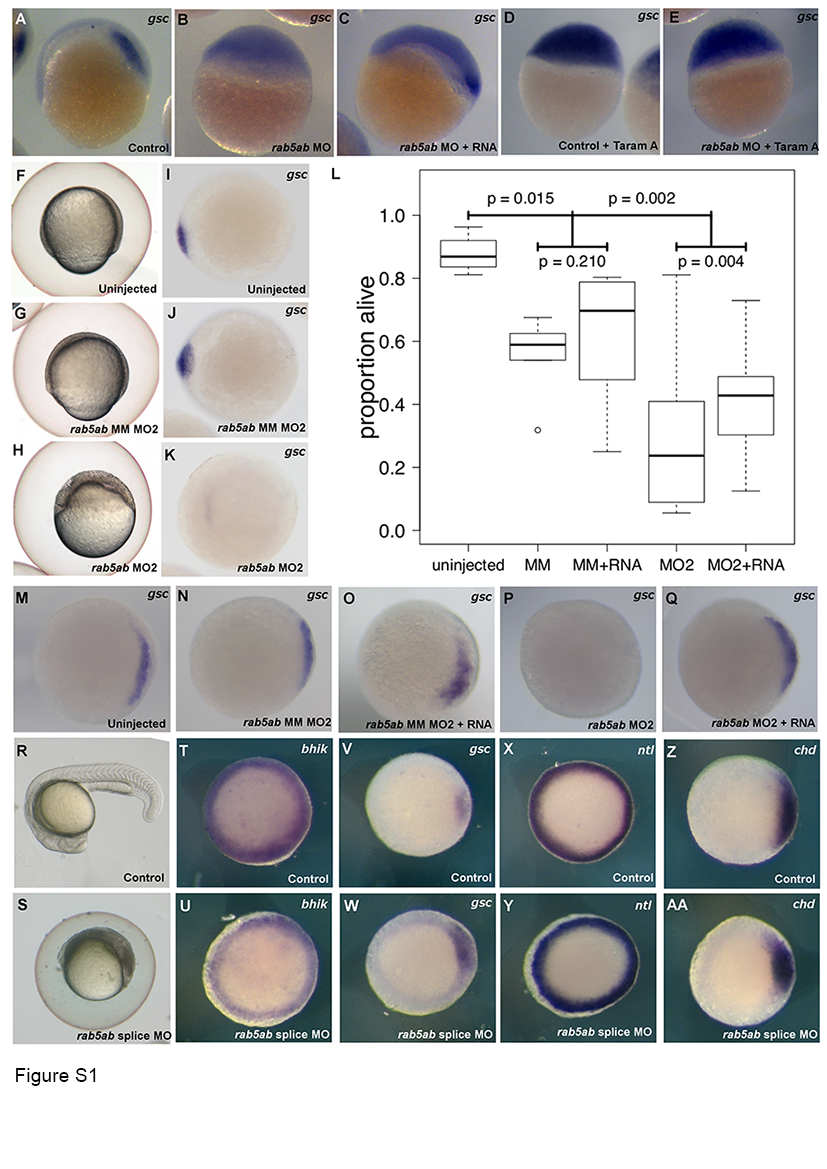

Supplement: Supplementary file 2 — Supplementary data Supplemental Fig. 1. Rescue of rab5ab MO׳s and phenotype of rab5ab splice MO. Lateral view of gsc expression in (A) control MO-injected embryos (n=30/30) compared to (B) rab5ab MO-injected embryos (n=30/30) and (C) rab5ab MO and rab5ab RNA injected embryos (n=30/31). Lateral view of gsc expression in (D) control and Taram-A RNA injected embryos (n=20/20) and (E) rab5ab morpholino and Taram-A RNA-injected embryos (n=20/20). Lateral view of (F) control uninjected embryos (n=62) (G) rab5ab mismatch MO2-injected embryos (n=119/119) and (H) rab5ab MO2 injected embryos when compared at the same time point (n=102/103). Animal view of gsc expression in 50% epiboly stage (I) control uninjected embryos (n=10/10) compared to (J) rab5ab mismatch (MM) MO2-injected embryos (n=10/10) and (K) rab5ab MO2 injected embryos (n=10/11). (L) Box-whisker plots show empirical distributions of uninjected, rab5ab MM MO2 injected, rab5ab MM MO2+RNA injected, rab5ab MO2 and rab5ab MO2+RNA injected embryos that survive to 30% epiboly. Horizontal lines denote median expression and boxes cover the interquartile range. Whiskers extend to 1.5 times the interquartile range, with additional outliers plotted as points. Animal view of gsc expression in 50% epiboly stage (M) control uninjected embryos (n=31/31) compared to (N) rab5ab MM MO2 injected embryos (n=51/51), (O) rab5ab MM MO2+RNA injected embryos, (P) rab5ab MO2 injected embryos (n=20/33) and (Q) rab5ab MO2+RNA injected embryos (n=36/42). Side view of (R) 24 hpf control embryo (n=30), side view of (S) embryos injected with 10 ng of rab5ab splice MO (n=30). Animal view of expression pattern of bhik in control (T) and rab5ab splice (U) morpholino injected embryos. Animal view of expression pattern of gsc in control (V) and rab5ab splice (W) morpholino injected embryos. Animal view of expression pattern of ntl in control (X) and rab5ab splice (Y) morpholino injected embryos. Animal view of expression pattern of chd in control (Z [file mmc1.zip › Fig S1_rev.tif]
